# Sociodemographic factors and social media use in 9-year-old children: the Generation R Study

**DOI:** 10.1186/s12889-021-12061-4

**Published:** 2021-10-30

**Authors:** Yueyue You, Junwen Yang-Huang, Hein Raat, Amy van Grieken

**Affiliations:** 1 The Generation R Study Group, Erasmus Medical Center, Rotterdam, The Netherlands; 2 Department of Public Health, Erasmus Medical Center, P.O. box 2040, 3000 CA Rotterdam, The Netherlands

**Keywords:** Sociodemographic inequality, Young children, Instant messaging, Social network site, Healthy social media use;

## Abstract

**Background:**

We aimed to investigate the associations between sociodemographic factors and instant messaging and social network site exposure among 9-year-old children.

**Methods:**

Data of 4568 children from the Generation R study, a population-based cohort study performed in Rotterdam, the Netherlands, were analyzed. Instant messaging exposure was defined as using online chat applications such as MSN, chat boxes, WhatsApp, and Ping. Social network site exposure was defined as using Hyves or Facebook. A series of multiple logistic regression analyses were performed, adjusting for covariates.

**Results:**

Children of low educated mothers had a higher odds ratio (OR) for instant messaging (OR: 1.44, 95% CI: 1.12, 1.86) and social network site exposure (OR: 1.73, 95% CI: 1.13, 2.66) than their counterparts. Being a child from a single-parent family was associated with instant messaging (OR: 1.48, 95% CI: 1.16, 1.88) and social network site exposure (OR: 1.34, 95% CI: 1.01, 1.78) more often than their counterparts. Children of low educated fathers (OR: 1.48, 95% CI: 1.12, 1.95) or from families with financial difficulties (OR: 1.28, 95% CI: 1.04, 1.59) were associated with a higher OR of social network site exposure than their counterparts.

**Conclusion:**

The findings suggest that several indicators of lower social position are associated with higher social network site and instant messaging exposure among 9-year-old children. More research is needed in younger children to understand the determinants and impact of social media use.

**Supplementary Information:**

The online version contains supplementary material available at 10.1186/s12889-021-12061-4.

## Introduction

Currently, using social media is among the most common activities of children and adolescents [1]. In general terms, social media can be defined as using any of the major social network sites (such as Facebook, Twitter, or Instagram), or communicating by instant messaging (such as MSN, chat boxes, or WhatsApp) [2]. A study by Perrin showed that, among children and adolescents aged 8 to 18 years old, social media use had increased from 10% in 2005 to 76% in 2015 [3]. Twenge et al. [4] reported that, among adolescents aged 16 years, 50% visited social network sites almost every day in 2008, and the percentage increased to 82% in 2016. Thus far, while research has focused on older children and adolescents (11 years and older), the use of social media is also rapidly increasing among younger children [5, 6].

Previous studies have indicated that social network site or instant messaging exposure may negatively affect children’s social, physical, and psychological aspects of development [7]. For example, a European study among children aged 11–16 years, showed that 30% of the children who spent more than 2 h per day on social network sites (Facebook and Twitter), reported one or more health issues, such as neglecting friends or sleeping less [8]. Grover et al. [9] reported that children who spent more time on instant messaging were less likely to have better academic performance. Therefore, identifying the factors that determine social network site and instant messaging exposure may help to guide future intervention development.

Although previous studies have focused on reporting on the social network site exposure, instant messaging has also proved to be highly popular with children [10]. What makes social network sites unique is not only that they allow individuals to meet strangers, but also that they enable users to articulate and make visible their social networks [11]. This can result in connections between individuals that would not otherwise have been made. On the other hand, for children or adolescents, instant messaging is a vehicle for communicating with friends and services and is primarily supported by friendship [12]. Thus, main purpose of using these accounts is to talk to friends rather than to develop relationships with people who have nothing to do with their daily social circle. This suggests that social network site exposure does not replace instant messaging exposure, and that certain factors may have different impacts on social network site and instant messaging exposure. Although few studies have investigated the associations between sociodemographic factors and social network site exposure among children aged 11 and older, the results are inconsistent [13, 14]. For example, some European studies have reported that children from parents with lower educational levels, or from households with low family income, or from an ethnic minority group use social network sites on a daily basis more often than their counterparts [15–17]. In contrast, another study in the U.S. reported that social network site use by children is not related to parental educational level nor to ethnic background [18]. Furthermore, studies on the associations between sociodemographic factors and instant messaging exposure are lacking [19]. In light of these inconsistencies and lack of research, it is necessary to understand which sociodemographic factors are associated with social network site and instant messaging exposure. Such understanding may provide relevant information for professionals as to which group of children is more likely to be exposed to social network sites and instant messaging. Accordingly, professionals might support these children to develop safe social media behavior. For example, child health professionals can work with families or schools to promote children’s understanding of the benefits and risks of social media.

The aim of this present study was to evaluate the associations between sociodemographic factors (parental educational level, parental employment status, net household income, financial difficulties, marital status, and child’s ethnic background) and instant messaging and social network site exposure. Cheng et al. [20] reported that sociodemographic characteristics are major factors that influence individual’s behaviors and health. Therefore, in this study, we focused on sociodemographic factors. The hypothesis was that children of lower educated parents, from lower household income, from families with financial difficulties, or from single-parent families had a higher odds ratio for instant messaging and social network site exposure .

## Material and methods

### Data source and study population

**Fig. 1:**
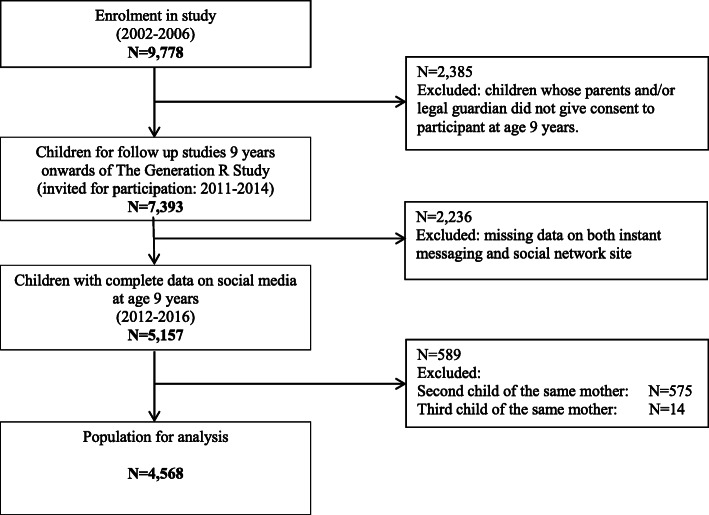
Flowchart of participants included for analysis

The present analyses used data from the Generation R study, which is a population-based, prospective cohort study from early fetal life onwards in Rotterdam, the Netherlands. The study is designed to identify early environmental and genetic causes and causal pathways leading to normal and abnormal growth, development, and health from fetal life, childhood and young adulthood. Midwives and obstetricians invited all pregnant women under their care with an expected delivery date between April 2002 and January 2006, living in the study area, and at the time of delivery to participate in the Generation R Study. In total, the cohort includes 9778 mothers and their children. For 7393 children informed consent was provided for data collection at age 9 years [21]. Children without data on both instant messaging and social network site exposure (*n* = 2236) were excluded. To avoid clustering of data, the second (*n* = 575) and third children (*n* = 14) of the same mother were excluded, leaving a study population of 4568 participants (Fig. 1). The Medical Ethical Committee of the Erasmus Medical Center, Rotterdam approved the study. Informed consent was obtained from a parent and/or legal guardian.

### Sociodemographic factors

Sociodemographic factors included maternal and paternal educational level, maternal and paternal employment status, net household income, financial difficulties, marital status, and child’s ethnic background. All related information was obtained via parental-completed questionnaire.

Maternal and paternal educational levels were obtained by questionnaire when the child was 6 years old and categorized as follows: high (university or Ph.D. degree), mid-high (higher vocational training), mid-low (> 3 years general secondary school, intermediate vocational training) and low (no education, primary school, lower vocational training, intermediate general school, or 3 years or less general secondary school) [22]. Maternal and paternal employment status (paid job and no paid job) and net household income (high: >€3300/month; middle: €2000–3300/month; low: <€2000/month) [23] were obtained by questionnaire when the child was 9 years old. Financial difficulties included having trouble paying for food, rent, and electricity bills in the past year. Parents received this description of financial difficulties and were asked “Did you have financial difficulties in the past years?”. Financial difficulties (yes/no) and marital status (married/cohabiting versus no partner) were obtained by questionnaire when the child was 9 years old. The child’s ethnic background was determined by the parents’ country of birth and was obtained by questionnaire when the child was 6 years old. If one of the child’s parents was born in another country other than the Netherlands, this country defined the child’s ethnic background. If both parents were born abroad, the mother’s country of birth prevailed. In accordance with the definitions by Statistics Netherlands, child’s ethnic background was divided into three categories: Dutch, other Western (American western, Asian western, European and Oceania), and non-Western (Indonesian, Cape Verdean, Moroccan, Dutch Antilles, Surinamese, Turkish, African, American non-western and Asian non-western). This operationalization is commonly used in studies among the Dutch population, thereby taking into account the different migrant groups living in the Netherlands [24].

### Instant messaging and social network site exposure

Instant messaging and social network site exposure were assessed by questionnaire when the child was 9 years old. Instant messaging exposure was defined as using online chat applications such as MSN, chat boxes, WhatsApp, and Ping. Parents received the definition of instant messaging and were asked “does your child use instant messaging?”. Social network site exposure was defined as using Hyves (a Dutch equivalent of Facebook) or Facebook. Parents received the definition of social network site and were asked “does your child use social network site?”

### Additional variables

Child age (years), sex (boy/girl), presence of siblings (yes/no), and season at measurement (spring, summer, fall, winter) were considered as potential confounders [13, 25]. This information was obtained by the questionnaire when the child was 9 years old.

### Statistical analysis

Descriptive statistics were used to describe the study population. The associations of sociodemographic factors with children’s instant messaging and social network site exposure were assessed using multiple logistic regression models. In the basic model, each indicator of sociodemographic factors was included separately, adjusting for these confounders: child age, sex, presence of siblings, and season at measurement. The full model was additionally adjusted for all sociodemographic factors (Bonferroni correction used for multiple logistic regression; corrected significance level is 0.05/8 = 0.006). Interactions between parental educational level (maternal and paternal educational level) and child’s ethnic background, parental employment status, net household income, and financial difficulties were assessed in the logistic regression models [26]. Collinearity between maternal educational level, paternal educational level, and income were assessed by pair-wise Spearman’s rho correlation coefficients. The correlation coefficient did not indicate collinearity (*r* > 0.6) between sociodemographic factors; therefore, these variables were included simultaneously in the full models. In total, 5 imputed datasets were calculated. If the primary interest is the point estimates and missing data is considered moderate, using 5–20 imputed datasets is considered appropriate [27]. In the current study, missing data ranged from 0 to 18.8%. Pooled odds ratios (ORs) and 95% confidence intervals (CIs) were calculated, and *p* < 0.05 was used to indicated statistical significance. All analyses were performed using IBM SPSS Statistics for Windows, version 24.0 Armonk, NY, USA: IBM Corp.

### Nonresponse analyses and interaction effects

Children with missing data on instant messaging and social network site exposure (*n* = 2236) were compared with children without missing data (*n* = 4568) using Chi-square tests. Data were more often missing for children from parents with a low educational level, parents without a paid job, a low household income, a family with financial difficulties, a single-parent family, or from a non-western ethnic background (all *p* < 0.05).

The interaction effects between parental educational level and child’s ethnic background, parental employment status, net household income, and financial difficulties are presented in Additional file 1: Appendix Table A. No statistically significant interaction terms were observed.

## Results

### Participants characteristics

Characteristics of the study population are presented in Table 1. In total, 31.6% of the mothers and 36.0% of the fathers had a high educational level. A little over half of the children were living in a high income household (55.7%), and approximately one-fifth of the families reported having financial difficulties (19.5%). The mean age of the children was 9.72 (SD: 0.32) years, and 50.4% were girls. Of all participants, 29.3% of the children were exposed to instant messaging, and 16.4% of the children were exposed to social network sites.

**Table 1:** General characteristics of the study population among 9-year-old children (*n* = 4568)

		Total n (%)	Missing n (%)
*Family characteristics*			
Maternal educational level	High	1309 (31.6)	426 (9.3)
	Mid-high	1199 (28.9)	
	Mid-low	1222 (29.5)	
	Low	412 (10.0)	
Paternal educational level	High	1369 (36.0)	767 (16.8)
	Mid-high	922 (24.3)	
	Mid-low	1000 (26.3)	
	Low	510 (13.4)	
Maternal employment status	Paid job	3098 (78.6)	628 (13.7)
	No paid job	842 (21.4)	
Paternal employment status	Paid job	3537 (95.3)	857 (18.8)
	No paid job	174 (4.7)	
Net household income^a^	>€3200/month	2415 (55.7)	234 (5.1)
	€2000–€3200/month	959 (22.1)	
	<€2000/month	960 (22.2)	
Financial difficulties	No	3631 (80.5)	55 (1.2)
	Yes	882 (19.5)	
Marital status	Married/cohabiting	3584 (86.9)	442 (9.7)
	No partner	542 (13.1)	
*Child characteristics*			
Gender	Girl	2304 (50.4)	0
	Boy	2264 (49.6)	
Age year, mean (SD)		9.72 ± 0.32	0
Siblings	Yes	3770 (83.4)	48 (1.1)
	No	750 (16.6)	
Ethnic background	Dutch	2796 (61.9)	54 (1.2)
	Other western	396 (8.8)	
	Non-western	1322 (29.3)	
*Season when completing questionnaire*			
	Spring	1282 (28.1)	0
	Summer	985 (21.6)	
	Autumn	1038 (22.7)	
	Winter	1263 (27.6)	
*Social media use*			
Instant messaging	Yes	1335 (29.3)	17 (0.4)
	No	3216 (70.7)	
Social network site	Yes	747 (16.4)	22 (0.5)
	No	3799 (83.6)	

*Note*: Table is based on non-imputed dataset

Values are presented as mean and standard deviation (SD) for continuous variables and frequencies with percentages (%) for categorical variables

^a^Net household income was not adjusted for household size

### Associations between sociodemographic factors and instant messaging and social network site exposure

Children of low educated mothers had higher odds of instant messaging (OR:1.29; 95%CI:1.01, 1.67) and social network site exposure (OR: 2.15; 95% CI: 1.59, 2.91) than children of high educated mothers (Table 2, Basic model). Results were comparable to paternal educational level. Children from low income household (OR: 1.61, 95% CI: 1.33, 1.96) or families with financial difficulties (OR:1.56, 95%CI:1.29, 1.88) had higher odds of social network site exposure than their counterparts. Finally, children from a single-parent family had higher odds of instant messaging (OR:1.43, 95%CI:1.15,1.78) and social network site exposure (OR:1.53, 95%CI:1.20,1.95) than children from a two-parent family.

**Table 2:** The associations between sociodemographic factors and instant messaging and social network site exposure among 9-year-old children (*N* = 4568)

	Instant messaging (yes)		Social network site (yes)	
	Basic model ^a^	Full model ^b^	Basic model ^a^	Full model ^b^
	OR (95% CI)	OR (95% CI)	OR (95% CI)	OR (95% CI)
Maternal educational level				
High	1.00	1.00	1.00	1.00
Mid-high	1.16 (0.97–1.39)	1.12 (0.93–1.36)	1.14 (0.89–1.47)	1.00 (0.76–1.32)
Mid-low	**1.50 (1.24–1.81)**	1.29 (0.85–1.96)	**1.92 (1.54–2.40)**	**1.58 (1.19–2.10)**
Low	**1.29 (1.01–1.67)**	**1.44 (1.12–1.86)**	**2.15 (1.59–2.91)**	**1.73 (1.13–2.66)**
Paternal educational level				
High	1.00	1.00	1.00	1.00
Mid-high	1.20 (0.98–1.49)	1.13 (0.91–1.40)	**1.42 (1.08–1.86)**	1.36 (0.94–1.96)
Mid-low	**1.39 (1.16–1.65)**	1.18 (0.95–1.47)	**1.90 (1.48–2.44)**	1.30 (0.98–1.71)
Low	**1.31 (1.03–1.66)**	1.08 (0.75–1.55)	**1.95 (1.49–2.56)**	**1.48 (1.12–1.95)**
Maternal employment status				
Paid job	1.00	1.00	1.00	1.00
No paid job	0.96 (0.78–1.17)	0.89 (0.71–1.13)	1.20 (0.99–1.47)	1.00 (0.80–1.24)
Paternal employment status				
Paid job	1.00	1.00	1.00	1.00
No paid job	1.14 (0.85–1.53)	1.09 (0.79–1.49)	1.14 (0.80–1.63)	0.94 (0.64–1.38)
Net household income†				
> €3200/month	1.00	1.00	1.00	1.00
€2000–€3200/month	1.12 (0.95–1.33)	1.09 (0.90–1.32)	**1.23 (1.00–1.51)**	0.92 (0.74–1.16)
< €2000/month	1.13 (0.95–1.34)	0.83 (0.66–1.04)	**1.61 (1.33–1.96)**	1.00 (0.74–1.34)
Financial difficulties				
No	1.00	1.00	1.00	1.00
Yes	1.13 (0.96–1.33)	1.06 (0.88–1.28)	**1.56 (1.29–1.88)**	**1.28 (1.04–1.59)**
Marital status				
Married/cohabiting	1.00	1.00	1.00	1.00
No partner	**1.43 (1.15–1.78)**	**1.48 (1.16–1.88)**	**1.53 (1.20–1.95)**	**1.34 (1.01–1.78)**
Ethnic background				
Dutch	1.00	1.00	1.00	1.00
Other western	1.07 (0.85–1.36)	1.11 (0.87–1.41)	0.80 (0.59–1.08)	1.23 (0.90–1.67)
Non-western	**1.24 (1.06–1.44)**	1.18 (0.99–1.40)	1.07 (0.89–1.28)	0.97 (0.69–1.37)

*Note*: Table is based on imputed dataset. Bold print indicates statistical significance

† Net household income was not adjusted for household size

Values represent odds ratios and 95% confidence intervals derived from (multiple) logistic regression analyses

^a^Each sociodemographic factor was added to the model separately, and model was adjusted for child age, gender, siblings and season of participation

^b^All sociodemographic factors were added to the model, and model was adjusted for confounders: child age, gender, siblings and season of participation

Using the Bonferroni correction in the multiple logistic regression (full model), the level of statistical significance is 0.05/8 = 0.006

After adjusting for all sociodemographic factors, independent associations were observed between maternal educational level and marital status with both outcomes (Table 2, full model). Children of low educated mothers had higher odds of instant messaging (OR: 1.44, 95% CI: 1.12, 1.86) and social network site exposure (OR: 1.73, 95% CI: 1.13, 2.66) than children of high educated mothers. Children living in a single-parent family had higher odds of instant messaging (OR: 1.48, 95% CI: 1.16, 1.88) and social network site exposure (OR: 1.34, 95% CI: 1.01, 1.78) than their counterparts. Children of low educated fathers showed an OR of 1.48 (OR: 1.48, 95% CI: 1.12, 1.95) with social network site exposure than their counterparts. Compared with children from families without financial difficulties, children from families with financial difficulties (OR: 1.28, 95% CI: 1.04, 1.59) were more likely to be exposed to the social network sites.

## Discussion

After adjustment for all sociodemographic factors, the results showed that children of low educated mothers and children from single-parent families were more likely to be exposed to instant messaging and social network sites. Children of low educated fathers or from families with financial difficulties were more likely to be exposed to social network sites.

Our study observed that 29.3% of the 9-year-old children were exposed to instant messaging and 16.4% of the children were exposed to social network sites. Given these findings, it is important for child health professionals to be aware of the fact that children start to use social media at a young age. Therefore, professionals may assist parents in talking to their children about their social media use as early as possible. Our study also contributes to the knowledge regarding the associations between sociodemographic factors and social media use (both instant messaging and social network site) in children of a young age. Several indicators of sociodemographic factors were found to be inversely associated with social media use. Future studies are needed, however, to shed more light on the social media use among younger aged children.

Previous studies have reported that, among children aged 14 and older, the maternal educational level was associated with using social media [15, 26]. Our study observed a similar finding in a younger age group (9-year-old). The results showed that children of low educated mothers were more likely to be exposed to instant messaging and social network sites than children of high educated mothers; likewise, compared to children of high educated fathers, children of low educated fathers were more likely to be exposed to social network sites. A possible explanation may be that adults (or parents) with low educational levels are more likely to use social media [28]. Therefore, their children may model this behavior. Low educated parents might be less aware of the possible adverse health effects due to social media use and, consequently, exert less control on their children’s social media use than high educated parents [16, 29]. Gentile et al. [30] showed that high educated parents were stricter in limiting the time their children spent on social media than low educated parents.

Children from single-parent families were more likely to be exposed to instant messaging and social network sites than children from two-parent families. This result is in line with previous studies [31, 32], which showed that children living in one parent households were more likely to use social media. A possible explanation for this finding could be that the number of adults at home is positively related children’s time interacting with them, which may displace time spent on social media [33, 34]. Moreover, single-parent families are known to experience more challenges to parenting in general [35], resulting in parents allowing their children more freedom to use media on their own rather than actively guiding their children’s media use [36]. For example, after working an entire day, parents often need some time to relax or rest, therefore, they may deliberately provide children with media sets (computer or iPad) in their bedrooms [37, 38].

Furthermore, children in our study living in families with financial difficulties were more likely to be exposed to social network sites than their counterparts. A cross-sectional study in Germany showed that children aged 8 to 12 years old who lived in families with financial difficulties were 2.1 times more likely to own a mobile phone [39]. Compared with children without a mobile phone, children with a mobile phone have more opportunities to access social media. Previous studies have indicated an association between a low socio-economic position and decreased child well-being [40]. Valkenburg et al. [41] reported that social network sites can help users in enhancing self-esteem and well-being. Therefore, children in families with financial difficulties maybe more likely to use social network sites to enhance happiness.

A major strength of this study is the availability of data on instant messaging as well as on social network site exposure among children as young as 9 years old, and on a broad range of sociodemographic factors. However, some limitations of the study have to be considered in the interpretation of the results. Firstly, this study is limited because the analysis of the data was cross-sectional, thereby preventing the testing of causal hypotheses. Future work should examine the associations longitudinally. Secondly, in this study, the information of instant messaging and social network site exposure was collected by the parent-reported questionnaire. Gentile et al. [30] reported a good validity of parent-reported the child social media use. For future studies, instant messaging and social network site exposure could be explored using objective measurement, for example, constructing exposure from the browsing history of users [42]. Furthermore, the variable net household income was not adjusted for household size because of a lack of information on household size. Adjustment for household size might create a more specific estimate of purchase power [43].

## Conclusion

The results of this study showed that a relatively high percentage of children already use social media at age 9 years. Children of low educated mothers and children from single-parent families, spent more time on social media as measured by instant messaging or social network site exposure. Children of low educated fathers or from families with financial difficulties were associated with social network site exposure. Health practitioners should be aware of the high usage of social media among young children. For intervention developers, findings in our study support the need to develop strategies for healthy social media use. We recommend future research in younger children to evaluate their instant messaging and social network site exposure, determinants of usage and associations with related health outcomes.

## Supplementary Information

**Additional file 1: Table A1.** *P*-values for interaction effects between parental educational level and child’s ethnic background, parental employment status, net household income, and financial difficulties on instant messaging and social network site exposure.

## Data Availability

The datasets generated and/or analysed during the current study are not publicly available due to individual privacy consideration, but are available from the data managers (datamanagementgenr@erasmusmc.nl) and Director Generation R, Vincent Jaddoe (v.jaddoe@erasmusmc.nl) after a written agreement about the use of the data made via the Technology Transfer Office of Erasmus MC.
