# Supplementary material for: Sociodemographic factors and social media use in 9-year-old children: the Generation R Study
Source: BMC Public Health. 2021 Oct 30;21:1966. doi: 10.1186/s12889-021-12061-4 (PMC8557565; doi:10.1186/s12889-021-12061-4)
Supplement: Supplementary file 1 — Additional file 1: Table A1. P-values for interaction effects between parental educational level and child’s ethnic background, parental employment status, net household income, and financial difficulties on instant messaging and social network site exposure. [file 12889_2021_12061_MOESM1_ESM.docx]

Table A1. P-values for interaction effects between parental educational level and child’s ethnic background, parental employment status, net household income, and financial difficulties on instant messaging and social network site exposure

| Item | Instant messaging exposure  p-value | Social network site exposure  p-value |
| --- | --- | --- |
| Maternal educational level × ethnic background | 0.465 | 0.106 |
| Maternal educational level × maternal employment status | 0.363 | 0.301 |
| Maternal educational level × net household income | 0.828 | 0.551 |
| Maternal educational level × financial difficulties | 0.174 | 0.223 |
| Paternal educational level × ethnic background | 0.965 | 0.673 |
| Paternal educational level × paternal employment status | 0.455 | 0.959 |
| Paternal educational level × net household income | 0.950 | 0.837 |
| Paternal educational level × financial difficulties | 0.441 | 0.114 |

Note: After applying Bonferroni correction for multiple testing (P=0.05/16=0.003), no statistically significant interaction effect was found.
